# Protective Effects of Dinitrosyl Iron Complexes under Oxidative Stress in the Heart

**DOI:** 10.1155/2017/9456163

**Published:** 2017-03-21

**Authors:** Valery I. Kapelko, Vladimir L. Lakomkin, Alexander A. Abramov, Elena V. Lukoshkova, Nidas A. Undrovinas, Asker Y. Khapchaev, Vladimir P. Shirinsky

**Affiliations:** Russian Cardiology Research and Production Complex, Ministry of Healthcare of the Russian Federation, 3rd Cherepkovskaya St., Building 15a, Moscow 121552, Russia

## Abstract

*Background*. Nitric oxide can successfully compete with oxygen for sites of electron-transport chain in conditions of myocardial hypoxia. These features may prevent excessive oxidative stress occurring in cardiomyocytes during sudden hypoxia-reoxygenation.* Aim*. To study the action of the potent stable NO donor dinitrosyl iron complex with glutathione (Oxacom®) on the recovery of myocardial contractile function and Ca^2+^ transients in cardiomyocytes during hypoxia-reoxygenation.* Results*. The isolated rat hearts were subjected to 30 min hypoxia followed by 30 min reoxygenation. The presence of 30 nM Oxacom in hypoxic perfusate reduced myocardial contracture and improved recovery of left ventricular developed pressure partly due to elimination of cardiac arrhythmias. The same Oxacom concentration limited reactive oxygen species generation in hypoxic cardiomyocytes and increased the viability of isolated cardiomyocytes during hypoxia from 12 to 52% and after reoxygenation from 0 to 40%. Oxacom prevented hypoxia-induced elevation of diastolic Ca^2+^ level and eliminated Ca^2+^ transport alterations manifested by slow Ca^2+^ removal from the sarcoplasm and delay in cardiomyocyte relaxation.* Conclusion*. The potent stable NO donor preserved cardiomyocyte integrity and improved functional recovery at hypoxia-reoxygenation both in the isolated heart and in cardiomyocytes mainly due to preservation of Ca^2+^ transport. Oxacom demonstrates potential for cardioprotection during hypoxia-reoxygenation.

## 1. Introduction

Oxidative stress in cells occurs when reactive oxygen species (ROS) prevail over the means of antioxidant protection. Reoxygenation after a period of hypoxia is a classic model of oxidative stress. Cardiomyocytes in hypoxic conditions mobilize a number of protective mechanisms to enable them to retain integrity in this critical period and restore normal function at reoxygenation. A deep fall in contractile function allows them to conserve limited stores of ATP and phosphocreatine. This is realized by means of limiting Ca^2+^ entry into cells [1] due to the shortening of action potential and early release of K^+^, which gradually reduces excitability of cardiomyocytes. Also, the activity of Ca^2+^-ATPase of sarcoplasmic reticulum (SERCA2) declines quite rapidly [2], with further reduction in Ca^2+^ uptake in the reticulum and subsequent release into sarcoplasm. However, an increased level of sarcoplasmic Ca^2+^ in diastole [3], especially during reoxygenation, induces the opening of the mitochondrial pores [4] with the subsequent fall of the potential.

In the last decade, many aspects of altered redox regulation in hypoxia became more evident. Although oxygen consumption during hypoxia is reduced, the mitochondria continue to generate superoxide [5], although in smaller amounts, about one-third of normal production, according to some estimates [1]. Perhaps, this is due to incomplete use of oxygen, because activation of NO synthases, especially of NOS3 [6] that occurs during the initial period of hypoxia, changes the competition between oxygen and nitric oxide for cytochrome oxidase in favor of the latter [7]. Moreover, NO, as well as S-nitrosothiols, can inhibit complex I by S-nitrosylation. This effect can be blocked by reducing agents, nitrolases and thioredoxin [8]. Thus, NO accumulation plays a protective role, as it allows chelating iron released from oxygen complexes to form NO-Fe^2+^ complexes in the presence of glutathione [9].

In light of these findings, elevated NO levels could potentially be useful during reoxygenation when the availability of oxygen to mitochondria suddenly multiplies. The well-known NO donors such as nitroglycerin, nitrosorbide, nitroprusside, and nitrite liberate NO in cells and tissues during their metabolism. However, some of these substances, such as sodium nitroprusside, contain cyanide anions, while other compounds including nitroglycerin can induce methemoglobinemia. As a result of decades-long research of professor Anatoly F. Vanin (Semenov Institute of Chemical Physics, Russian Academy of Sciences) and his colleagues, it was established that S-nitrosothiols present in cells can easily interact with iron ions released from the iron donor ferritin to form dinitrosyl iron complexes (DNIC) [10, 11]. The main feature of DNIC is that they serve as carriers of the “ready-to-use,” iron-stabilized form of NO and do not contain toxic components. When local NO concentration decreases the DNIC release nitric oxide and chelate it while in excess, thus functioning as nitric oxide depot/buffer in cells.

This concept was embodied at the Russian Cardiology Research and Production Complex by creating a drug Oxacom (Patent RU2291880) which represents a DNIC with glutathione as a ligand. In preclinical studies, a dose-dependent hypotensive effect of Oxacom in conscious rats was observed [12]. Oxacom injection substantially increased nitric oxide content in various rat organs. Lungs and liver demonstrated 5-6-fold increase of NO content while in the heart NO levels rise 14-fold [13]. During phase 1 and 2 clinical trials Oxacom was tested in healthy volunteers and in patients with hypertensive crisis. In these groups, it consistently reduced arterial blood pressure by 15–20% for 8–10 hours [14]. In the present report, we provide evidence that DNIC in the form of Oxacom could be successfully used to protect cardiomyocytes from functional decline and damage associated with hypoxia-reoxygenation and oxidative stress. These results for the first time outline the novel possible therapeutic implication for Oxacom as antihypoxic cardioprotective drug.

## 2. Materials and Methods

### 2.1. Isolated Heart Experiments

Hearts were isolated from male Wistar rats (*n* = 48) anesthetized with ketamine (100 mg/kg). The protocol of experiments was approved by the Animal Ethics Committee of the Institute of Experimental Cardiology of the Russian Cardiology Research and Production Complex (# 3/15). Hearts were isolated from rats anesthetized with ketamine (100 mg/kg). Hearts were cannulated and coronary vessels were retrogradely perfused at 37°C using standard Krebs-Henseleit solution containing 11 mM glucose and presaturated with carbogen (95% O_2_, 5% CO_2_). A latex balloon was introduced into the left ventricle (LV) and isovolumic pressure in LV and ECG were monitored. The balloon was filled with liquid to set diastolic pressure at 10–12 mm Hg in order to achieve an optimal distension of LV. Retrograde perfusion was conducted at constant pressure of 70 mm Hg. Electrodes were placed on the right atrium for electrostimulation.

Heart perfusion was performed in a specialized Hugo Saks perfusion system. The LV pressure and ECG were recorded using Harvard Apparatus (USA) sensors and amplifiers. All signals (LV pressure and coronary perfusion rate) were transferred to the computer through the preliminary amplifiers and analog-to-digital converter (ADC:USB-6215, National Instruments, USA, using sampling rate of 1000 Hz). In addition to analog signals (maximum and minimum LV pressure, rates of pressure rise and drop, and heart rate) the heart work index (the product of developed pressure and heart rate), which characterizes the overall energy consumption, and relaxation index (LV −*dP*/*dt* divided by LV developed pressure) were calculated.

In order to manage the ADC and record primary signals as binary files on the computer's hard drive, as well as for further analysis of signals, computer programs were developed by Dr. Lukoshkova using National Instruments LabVIEW graphical programming environment. These programs calculate functionally relevant parameters in each cardiocycle, average these parameters for every 2 seconds, record all estimated parameters into text files, and continuously display averaged values as trends on the monitor screen.

The program is designed for the analysis of records, primarily, of calculated values. It allows examine trends of functionally relevant parameters throughout the experiment and at any stage of the experiment. It provides options to remove artefact values and average the results of measurements in a variety of modes: (a) using averaging intervals from 6 s to 30 min; (b) linking the beginning of an averaged segment to the mark introduced by the operator during the experiment; (c) averaging between marks; and (d) combining averaging modes. The results of calculations can be written as text files or directly transferred to Excel spreadsheet using a ClipBoard function.

#### 2.1.1. Experimental Protocol

After recording of the cardiac baseline function, stepwise electric stimulation with increasing rate exceeding the spontaneous rate was applied using increments of 0.5 Hz up to 12 Hz and pacing for 15 sec at each rate. Then electrostimulation was stopped and 10 minutes later the functional parameters were recorded before the start of hypoxic perfusion. The 30 min hypoxia (replacement of O_2_ by N_2_ in perfusate) was followed by 30 min reoxygenation. This protocol was chosen after the preliminary tests, which revealed that 20-minute hypoxia was insufficient to cause cardiac dysfunction and the degree of recovery was close to 100%. At the end of experiment, electric stimulation with increasing rate was repeated. Oxacom (30 nM) was added to perfusate during hypoxic perfusion and it was not added at reoxygenation.

An assessment of the heart dysfunction was performed by comparison of its functional parameters before, during, and after hypoxia. An indispensable component of this evaluation was the magnitude of LV diastolic pressure, which reflects the degree of myocardial stiffness occurring during hypoxia.

### 2.2. Determination of Glutathione Levels in Myocardial Tissue

Rat hearts were washed with phosphate buffered saline and frozen in liquid nitrogen immediately after experimental procedures. The tissue was ground in liquid nitrogen, and three volumes of 5% 5-sulphosalicylic acid (SSA) were added to 0.1–0.3 mg of the powder. The tissue sample was extracted using a glass-glass homogenizer on ice and centrifuged at 10,000 ×g for 10 min at 4°C to pellet the proteins. The supernatant fraction (up to 10 *μ*L) was used for measurements of total glutathione (GSH + GSSG) content using Glutathione Assay Kit (Sigma) according to the manufacturer's instructions in a buffer containing 95 mM KH_2_PO_4_, pH 7.0, 0.95 mM EGTA, 48 mM NADPH, 0.031 mg/mL 5,5′-dithiobis(2-nitrobenzoic acid) (DTNB), 0.115 U/mL glutathione reductase, and 0.24% SSA in a final volume of 200 *μ*L. Kinetics of DTNB reduction to 5-thio-2-nitrobenzoic acid (TNB) were measured using a Victor X3 plate reader (Perkin Elmer) at 405 nm. The amount of glutathione was calculated using a standard curve of reduced glutathione. Data were expressed as a mean from at least triplicate measurements ± standard deviation (SD). Amount of reduced glutathione (GSH) was calculated from measurements where both NADPH and glutathione reductase were excluded from the assay mixture. Average relative amount of reduced glutathione in the heart was calculated based on determined values.

### 2.3. Isolation and Manipulation of Cardiomyocytes

Right before cardiomyocyte isolation all buffers were supplemented with 1000 U/L heparin, warmed to 37°C in a water bath, and bubbled with carbogen (95% O_2_, 5% CO_2_) for 30 min. Rat heart was cannulated through aorta and perfused for 10 min according to the method of Langendorf to remove blood with Krebs-Henseleit buffer supplemented with 1.2 mM Ca^2+^. Next, heart was perfused with Ca^2+^-free buffer for 30 min to remove Ca^2+^ from extracellular spaces. After washing steps, heart was perfused in a recirculation mode for 30 min with 10 mL of 0.5 mg/mL collagenase (CLS 2, Worthington, 325 U/mg) solution in a Ca^2+^-free buffer. Following perfusion the heart was placed in a petri dish with 10 mL of fresh collagenase solution and minced in 1-2 mm^3^ pieces using fine scissors. Tissue suspension was transferred in 50 mL tube and gently pipetted for 10–15 min using sterile 5 mL plastic pipette with wide opening. Digested tissue was filtered through the 200 micron Nylon mesh to separate isolated cardiomyocytes from undigested heart fragments. Cardiomyocyte suspension was left in a sterile 50 mL tube for 10 min to settle down live cells. Then the supernatant was removed and cardiomyocytes were gently resuspended in 10 mL of albumin solution (6 mg/mL) supplemented with 0.1 mM Ca^2+^ and left for 10 min to settle. This step was repeated several times by placing cells in an albumin solution with increasing concentration of Ca^2+^ using steps of 0.25 mM until 1 mM Ca^2+^ was set. Cardiomyocytes were kept at 4°C in Krebs-Henseleit solution supplemented with 1 mM Ca^2+^ and 6 mg/mL albumin no more than 12 hours after isolation.

In order to reproduce normoxic or hypoxic conditions during the experiment cardiomyocytes were placed in experimental chamber in the buffer bubbled with carbogen or hypoxic gas mixture (95% N_2_, 5% CO_2_), respectively, and the chamber space above the buffer was constantly flashed with the corresponding gas mixture.

### 2.4. Ca^2+^ Transient Measurements in Cardiomyocytes

Cardiomyocytes were loaded with fluorescent Ca^2+^ indicator Fluo-4 (Invitrogen) for 20 min in the dark and transferred in a 250 *μ*L experimental chamber made of plastic walls and glass coverslip as the bottom. Two platinum electrodes are fixed at the opposite walls of the chamber. These electrodes are connected to Grass SD9 electrostimulator and are used to pace cardiomyocytes in the chamber by rectangular electric impulses at 38 V and 1 Hz. The chamber is mounted on the stage of an inverted AxioVert 200 M microscope (Zeiss) in a thermostat set at 37°C. Buffer is delivered in the chamber by gravity flow at 1–5 mL/min and removed from the chamber using a peristaltic pump. Cardiomyocytes are monitored through the coverslip using x63 oil objective and AxioCam HS high speed CCD camera (Zeiss). Fluorescence of Fluo-4 is excited using an HBO 103W/2 mercury lamp (Osram) and an appropriate filter cube for FITC-based fluorophores. Illumination intensity is kept low and exposure times are made short in order to prevent Fluo-4 bleaching and cardiomyocyte damage. Fluorescent signal from the individual cardiomyocytes is recorded at 50–200 frames/sec and images are streamlined to a terabyte hard drive for further processing using AxioVision Physiology software (Zeiss).

### 2.5. Reactive Oxygen Species Measurement in Cardiomyocytes

Fluorescent indicator Dihydrorhodamine 123 (Thermo Fisher Scientific) was used to assess reactive oxygen species (ROS) accumulation in isolated rat cardiomyocytes. The same experimental setup was used as described above for Ca^2+^-transient measurements in cardiomyocytes. Cells were loaded with DHR 123 for 20 min. Electrostimulation of cardiomyocytes was done in parallel with fluorescence recording and between interventions cells were kept in the dark.

### 2.6. Statistics

Statistical processing of experimental data obtained in isolated heart and isolated cardiomyocyte studies was performed using Statistics Package in Microsoft Excel 2013 and Student's *t*-test. Values in the tables and text are given as *M* ± SEM unless indicated differently.

## 3. Results and Discussion

### 3.1. Isolated Perfused Heart Experiments

#### 3.1.1. Selection of Oxacom Concentration

In order to determine an optimal concentration of Oxacom to be used during hypoxic perfusion, we studied this compound across the concentration range of 0.01–2.7 *μ*M in oxygenated hearts. The coronary flow rate was monitored as the main parameter as the increased rate under constant perfusion pressure indicates a decreased tone of the coronary vessels. Coronary flow rate clearly increased from 16.0 ± 1.2 to 20.2 ± 1.2 mL/min, but the changes in LV developed pressure were modest, about +7–9%. Only Oxacom concentration of 2.7 *μ*M caused a distinct decrease in LV developed pressure by 24%. In this regard, for further work the concentration of Oxacom 30 nM was selected, which reliably increased coronary flow rate and the index of relaxation by 8-9%. Both these effects could be interpreted as facilitation of Ca^2+^ removal from smooth muscle cells and cardiomyocytes, the events compatible with the reduction of hypoxic injury to myocardium.

#### 3.1.2. Hypoxia-Reoxygenation in Control Experiments

Baseline cardiac functional parameters are important for recovery of function after hypoxia-reoxygenation. It is known that lower intensity of oxidative metabolism at the beginning of hypoxia provides better recovery after reoxygenation. In this regard, the experiments where LV developed pressure was below 130 mm Hg and heart rate was below 230 beats/min were excluded from analysis.

Hypoxic perfusion of the heart resulted in a steep drop of LV developed pressure already in the first minutes of hypoxia along with increasing LV diastolic pressure that characterized myocardial contracture. Cardiac arrhythmias were observed in hypoxic period and they increased dramatically during reoxygenation. Arrhythmic events included extrasystoles, idioventricular rhythm, and fibrillation, which ceased spontaneously. Altogether, due to these reasons 4 hearts of 15 total did not restore function at the end of experiments.

By the end of reoxygenation the heart rate restored function completely while LV developed pressure restored function only by 27 ± 6% (by 34 ± 4% excluding experiments with zero recovery). The cardiac work changed accordingly. The LV diastolic pressure dramatically rose during hypoxia and slightly increased by the end of reoxygenation averaging 57 ± 5 mm Hg.

#### 3.1.3. Action of Oxacom in Hypoxia-Reoxygenation

**Figure 1:**
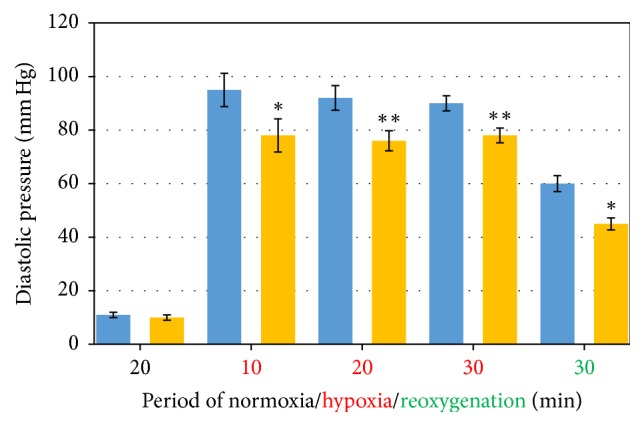
LV diastolic pressure after periods of normoxia (20 min), hypoxia (10–20–30 min), and reoxygenation (30 min) in the absence (blue) and in the presence of Oxacom (orange) in hypoxic perfusate. ^*∗*^*p* < 0.05; ^*∗∗*^*p* < 0.01.

During 10–30 min period of hypoxia in the presence of Oxacom the plateau level of LV diastolic pressure was consistently lower by 12–20 mm Hg than in control and this difference was statistically significant (Figure 1). During reoxygenation step, this difference persisted throughout the period. The rate of LV diastolic pressure lowering was higher in these experiments than in controls, especially at the initial stage of reoxygenation. Thus, Oxacom attenuated both hypoxia- and reoxygenation-induced myocardial contractures.

In both groups studied, the cardiac functional parameters before hypoxia were similar (Table 1). After hypoxia-reoxygenation the average values in Oxacom group tended to be better whereas −*dP*/*dt*max parameter was significantly higher than in control.

Recurrent arrhythmias were observed during both periods of hypoxia and reoxygenation in control and Oxacom experiments. Overall, the arrhythmias were observed in 10 out of 11 experiments in control group and in 7 out of 9 experiments in Oxacom group. The average integral length of arrhythmic events in control group was 14 ± 2 min, half of which originated from ventricular tachycardia (7 ± 1 min). In Oxacom group, the same distribution of arrhythmic events was observed; however, the values were significantly smaller, 4 ± 1 min and 2 ± 1 min (*p* < 0.02).

First NO-related effects on hypoxia- and reoxygenation-induced arrhythmias, especially ventricular arrhythmias, were reported in the 1970s when nitroglycerin was shown to be effective in these conditions (for review see [15]). Later several groups used NO precursor L-arginine as well as L-arginine methyl ester to cope with experimental ventricular arrhythmia [16, 17]. Although these studies reported successful prevention of arrhythmia, the concentrations of substances used to achieve restoration of normal electric activity of the heart ranged from 5 mM to 100 mM exceeding estimated concentration of L-arginine in plasma 50–1000-fold [18].

**Figure 2:**
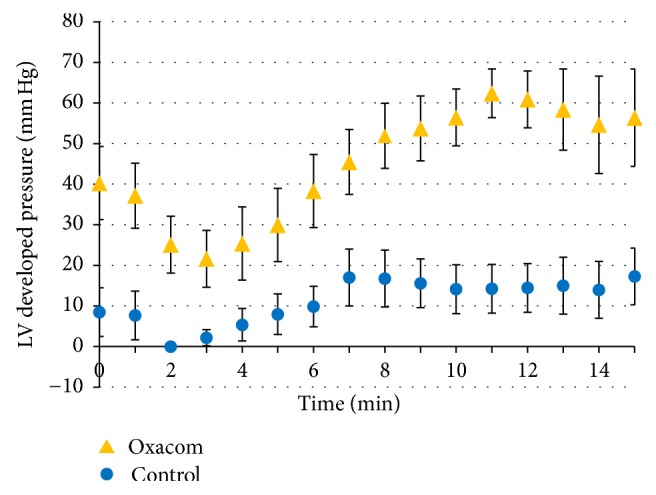
The time course of LV developed pressure during reoxygenation in groups in the absence and in the presence of Oxacom in hypoxic perfusate. Values in Oxacom group are significantly different from control group (*p* < 0.05).

The time course of LV developed pressure highly depended on arrhythmias because, during arrhythmic episodes, it dropped almost to zero. Still, in the presence of Oxacom LV pressure was maintained at higher levels throughout the hypoxic period and especially at the start of reoxygenation when arrhythmias in Oxacom group quickly ceased (Figure 2). The developed pressure in Oxacom group was also higher throughout reoxygenation period comprising 29 ± 4% of the initial value at 10 min (control 8 ± 4%, *p* < 0.01), 34 ± 6% at 20 min (control 15 ± 5%, *p* < 0.05), and 41 ± 3% at 30 min (control 33 ± 4%, *p* < 0.01, paired Student's *t*-test).

An attempt to restore cardiac function using L-arginine was undertaken by Agulló et al. [19]. They added 3 mM L-arginine to a perfusing solution for isolated hearts and improved recovery of the contractile function after reoxygenation from 2% to 12% of the initial value. An opposite effect was noticed when an inhibitor of soluble guanylate cyclase was used. Thus, the protective effect of arginine was apparently realized through the increased formation of cGMP.

**Figure 3:**
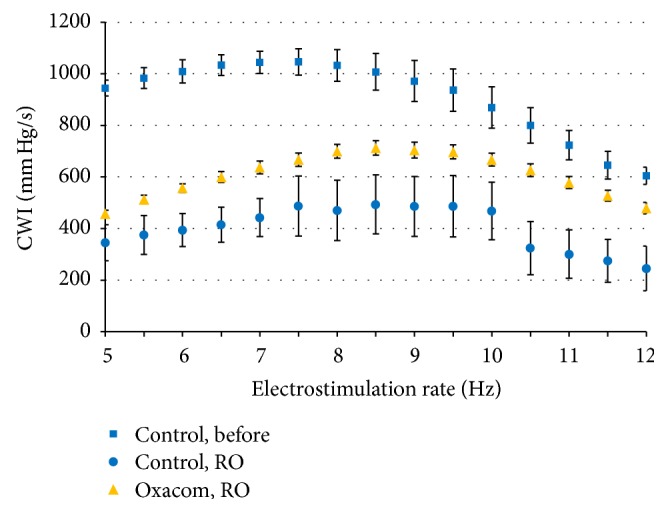
Dependence of cardiac work index (CWI) on the rate of heart electrostimulation before and after hypoxia-reoxygenation. CWI = LV developed pressure × heart rate; RO, reoxygenation. Difference between Oxacom and control after reoxygenation is statistically significant at 6-7 and 10–12 Hz (*p* < 0.05).

Electrostimulation with increasing rate was performed before and after hypoxia-reoxygenation. The results shown in Figure 3 demonstrate that stimulation of normoxic hearts at the rate of 5.0−7.5 Hz was accompanied by the rise in cardiac work index. This increase was rather small due to the high levels of developed pressure in the group (183–195 mm Hg). At higher stimulation rates, the index declined smoothly; still, all the hearts successfully reproduced the highest rate of 12 Hz.

After reoxygenation cardiac work index in control group stimulated at initial 5 Hz rate was significantly lower than in Oxacom group (*p* < 0.02). Further increase of stimulation rate resulted in the failure of 3 out of 11 control hearts to reproduce the frequency above 10 Hz while two hearts failed at 8 Hz. In contrast, all the hearts in Oxacom group successfully reproduced the highest applied stimulation rate. Based on the heart performance, the average cardiac work index in Oxacom group was 1.5–2-fold higher than in control group (*p* < 0.01). At maximal rate of stimulation, the cardiac work index in Oxacom group accounted for approximately 75% of the CWI for untreated normoxic heart.

Thus, addition of 30 nM Oxacom to hypoxic perfusate reduced hypoxic and reoxygenation contracture, maintained steady cardiac work during the period of hypoxia, and increased the degree of functional recovery of the heart following reoxygenation. In addition, Oxacom supported the ability of the hearts to reproduce the highest rate of stimulation and significantly increased the LV developed pressure at any frequency compared to control group not exposed to this compound. It should be pointed out that the beneficial effect of Oxacom was realized at 30 nM while similar effects of L-arginine and L-arginine methyl ester were achieved at 3 mM–100 mM [16, 17, 19], that is, at several orders of magnitude higher concentrations.

### 3.2. Reduced Glutathione Content in the Hearts Subjected to Hypoxia-Reoxygenation

Reduced glutathione in control normoxic hearts comprised 79.9 ± 2.1% of the total glutathione whereas after hypoxia-reoxygenation it decreased to 62.1 ± 1.1% that is, by 22%. In the hearts subjected to hypoxia-reoxygenation in the presence of 30 nM Oxacom the level of GSH was 58.3 ± 1.5%. Thus, low dose of Oxacom did not significantly alter the content of GSH in our model of hypoxia-reoxygenation.

### 3.3. Effects of Oxacom on Ca^2+^ Transients in Isolated Rat Cardiomyocytes Subjected to Hypoxia-Reoxygenation

**Figure 4:**
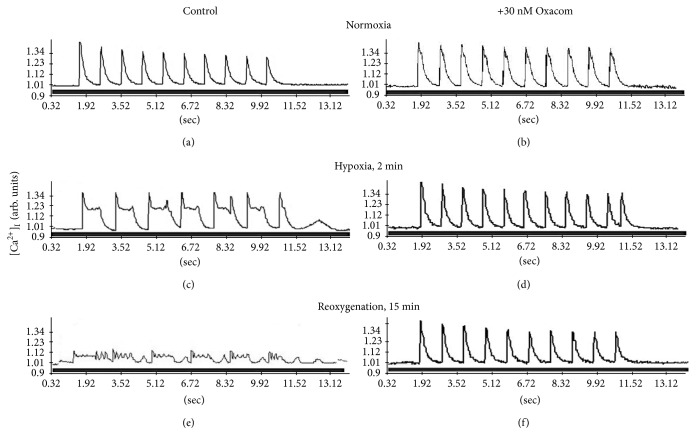
Effects of Oxacom on Ca^2+^ transients in isolated adult rat cardiomyocytes subjected to hypoxia-reoxygenation. Cardiomyocytes loaded with Fluo-4 were preincubated with 30 nM Oxacom for 30 min and electrostimulated by ten rectangular pulses at 1 Hz under conditions of normoxia, after 2 min of hypoxia produced by exchanging regular perfusion buffer saturated with carbogen (95% O_2_, 5% CO_2_) for hypoxic buffer saturated with 95% N_2_ and 5% CO_2_ and then after 15 min of reoxygenation. Typical recordings are shown. The legend for ordinate on the left applies to all graphs.

Control cardiomyocytes electrically paced at 1 Hz under normoxic conditions respond by the regular Ca^2+^ spikes produced simultaneously in all regions of sarcoplasm and generate no Ca^2+^ transients in the absence of electrostimulation (Figure 4). The shape of Ca^2+^ peaks is asymmetric with the steep upward shoulder and more shallow downward shoulder especially in its lower part. This shape of Ca^2+^ transient reflects molecular events underlying Ca^2+^ transport in cardiomyocytes. Fast rise in sarcoplasmic Ca^2+^ is achieved through the opening of Ca^2+^ channels and fast diffusion of Ca^2+^ ions in the sarcoplasm along the gradient. The reverse process of Ca^2+^ removal from the sarcoplasm into Ca^2+^ stores and across the sarcolemma outside the cell works against Ca^2+^ gradient and requires energy. This task is mainly fulfilled by Na/Ca exchanger (NCX) and Ca-ATPases such as SERCA2 and PMCA in cardiomyocytes. Working in direct mode NCX expels one Ca^2+^ ion from the sarcoplasm into extracellular space in exchange for three Na^+^ ions using electrochemical gradient for Na^+^ across sarcolemma generated by Na/K-ATPase. NCX has low affinity and high capacity for Ca^2+^ and is mostly active at peak Ca^2+^ concentration in the sarcoplasm. SERCA2 and PMCA have higher affinity and lower capacity for sarcoplasmic Ca^2+^ and are more effective at moderate to low Ca^2+^ concentrations.

Under normoxic conditions NCX and ion pumps promptly remove Ca^2+^ from the sarcoplasm to achieve its basal levels before the next electric stimulus arrives. The total length of Ca^2+^ transient under control normoxic conditions is 200–250 ms. During hypoxia the peak levels of free ionized Ca^2+^ entering cardiomyocytes modestly increase judging by the increase of Ca^2+^ transient amplitude (Figure 4(c)). At the same time the process of Ca^2+^ sequestration is retarded which is reflected by a “dome” on the downward shoulder of Ca^2+^ peak and about 1.5-fold increase in time required to reach basal Ca^2+^ level. Still, the initial removal of high Ca^2+^ from hypoxic cardiomyocytes is not altered in our model and the level of Ca^2+^ declines swiftly as in control cells suggesting that NCX function may not be critically affected in these conditions unlike the function of SERCA2 and PMCA. Additionally, cardiomyocytes become partially refractory and do not respond to every electric stimulus. Based on the “area under the curve” measurements using ImageJ software (NIH, USA) the average content of free ionized Ca^2+^ per Ca^2+^ peak is 3-fold higher in hypoxic than in normoxic cardiomyocytes indicating substantial Ca^2+^ overload. Noteworthily, in our experimental model cardiomyocytes hardly recover from more than 2 min of hypoxia and subsequent reoxygenation after this period of time does not improve their Ca^2+^ transients indicating irreversible damage to the molecular systems of Ca^2+^ transport (Figure 4(e)).

**Figure 5:**
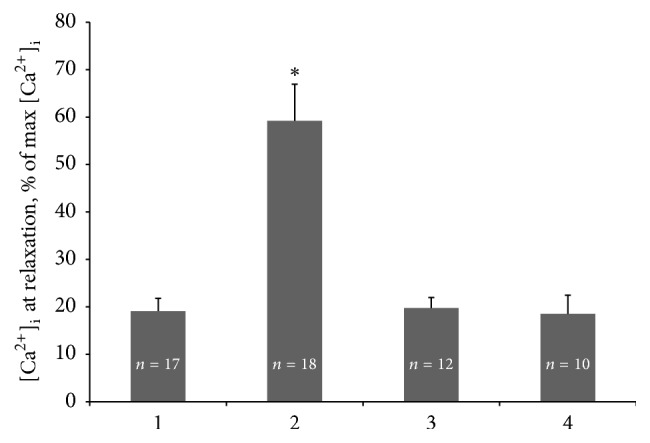
Quantitation of Oxacom effects on Ca^2+^ levels at relaxation in isolated adult rat cardiomyocytes subjected to hypoxia-reoxygenation. 1, control (normoxia); 2, hypoxia, 2 min; 3, hypoxia + 30 nM Oxacom, 2 min; 4, reoxygenation + 30 nM Oxacom, 15 min. *n*, number of cells for each condition. Mean ± SD. ^*∗*^*p* < 0.01, 2 versus 1, 3, and 4.

In contrast, these negative events are not developed when cardiomyocytes are preincubated with 30 nM Oxacom and this compound is present in the perfusate throughout the experiment (Figures 4(b), 4(d), and 4(f)). Oxacom does not alter Ca^2+^ transients in normoxic perfusion conditions and maintains normal parameters of Ca^2+^ peaks under both hypoxic and reoxygenation conditions. In the presence of Oxacom cardiomyocytes respond to each of ten electric stimuli in a packet by a single Ca^2+^ spike demonstrating no refractory behavior.  Figure 5 shows the quantitative assessment of basal Ca^2+^ in rat cardiomyocytes averaged from the set of experiments such as those presented in Figure 4. It confirms that the level of basal Ca^2+^ in cardiomyocytes subjected to 2 min of hypoxia is 3-fold higher than in normoxic control cells and in cardiomyocytes preincubated with 30 nM Oxacom and subjected to hypoxia-reoxygenation. Because of the relatively small contribution of excessive extracellular Ca^2+^ entry in our model of acute hypoxia of cardiomyocytes we favor the hypothesis that observed Ca^2+^ overloading is mainly due to the failure of Ca^2+^ sequestration systems. In separate experiments, we found that 0.3 nM Oxacom was able to reproduce the effects of 30 nM Oxacom on Ca^2+^ transients in isolated cardiomyocytes subjected to hypoxia-reoxygenation (see supplemental figures S1 and S2 in Supplementary Material available online at https://doi.org/10.1155/2017/9456163). However, such low dose of Oxacom was not effective in isolated perfused heart model.

Thus, Oxacom prevents alterations induced by hypoxia-reoxygenation in Ca-ATPases and, perhaps, NCX and allows cardiomyocytes maintain normal Ca^2+^ transport in these stressful conditions.

### 3.4. Effects of Oxacom on Cardiomyocyte Contractility and Viability

Contractile activity of cardiomyocytes, based on their visual assessment under the microscope, was fully consistent with the parameters of Ca^2+^-transport; each Ca^2+^ peak was followed by a single contraction of cellular body. Prolonged Ca^2+^ elevations in hypoxia were accompanied by the same lengthy cellular contractions. Thus, the effect of Oxacom on contractile activity of cardiomyocytes in our model was mainly determined by its impact on the process of Ca^2+^ removal from the sarcoplasm.

**Figure 6:**
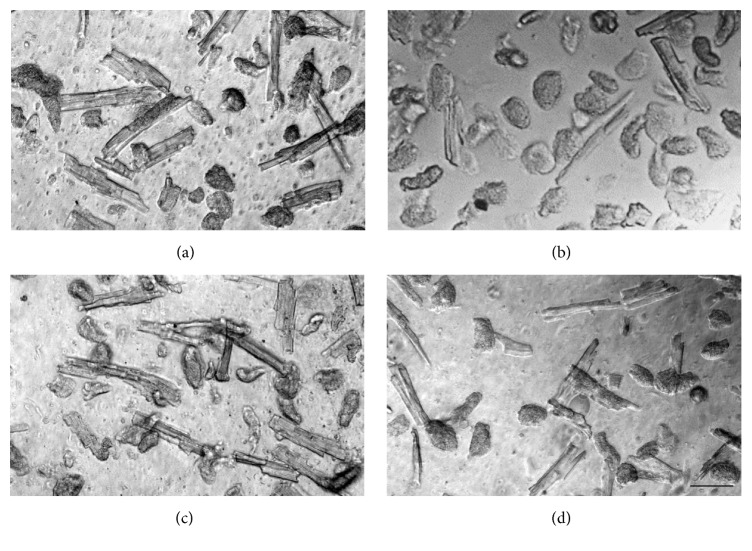
Effects of Oxacom on rat cardiomyocyte viability following hypoxia and reoxygenation. (a) Freshly isolated cardiomyocytes in normoxic buffer; (b) after 2 min of hypoxia; (c) after 2 min of hypoxia in the presence of 30 nM Oxacom; (d) after 15 min of reoxygenation in the presence of 30 nM Oxacom. Phase contrast. Bar, 50 *μ*m.

Oxacom improved viability of cardiomyocytes subjected to hypoxia-reoxygenation. Our isolation procedure typically yields about half of viable cardiomyocytes in the total cell isolate. These cells display characteristic elongated shape with sharp edges (Figure 6(a)). After two minutes of hypoxia elongated live cardiomyocytes comprised about 12% whereas other cells were rounded and supercontracted exhibiting irreversible damage (Figure 6(b)). Preincubation of cardiomyocytes with 30 nM Oxacom in normoxic conditions does not change the appearance of cell sample that contained 53% of viable cells.

Exposure of cardiomyocytes to 2 min hypoxia in the presence of Oxacom resulted in 52% elongated cells in the sample (Figure 6(c)) similar to normoxic control. After reoxygenation in the presence of Oxacom more that 40% of cardiomyocytes were viable (Figure 6(d)) while there were no viable cells in control sample. Similar results were obtained using quantitation of cells stained with Trypan Blue (data not shown). Thus, low doses of Oxacom protect isolated cardiomyocytes from profound damage and death caused by hypoxia-reoxygenation.

### 3.5. Effects of Oxacom on Reactive Oxygen Species Accumulation in Isolated Rat Cardiomyocytes Subjected to Hypoxia-Reoxygenation

**Figure 7:**
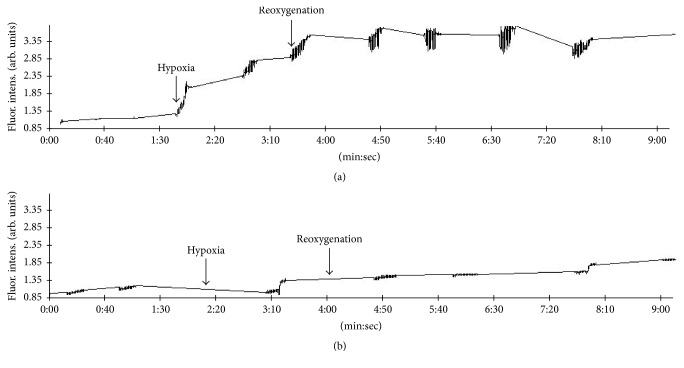
Effects of Oxacom on reactive oxygen species accumulation in adult rat cardiomyocytes subjected to hypoxia-reoxygenation. Cardiomyocytes loaded with DHR-123 fluorescent indicator for ROS were sequentially perfused with normoxic and hypoxic buffers (indicated by arrows) and electrostimulated by rectangular pulses as 1 Hz. “Noisy” fragments of the curve correspond to electrostimulation and fluorescence recording periods. (a) Control cardiomyocytes. (b) Cardiomyocytes preincubated with 30 nM Oxacom. Typical recordings are shown.

The alterations of ion-transporting molecular systems of cardiomyocytes in conditions of hypoxia-reoxygenation and ischemia-reperfusion are often linked to the damage of ion transporters and regulatory proteins by reactive oxygen species (ROS) that accumulate in cells during the disturbances in oxygen supply. As shown in Figure 7, ROS levels are not significantly altered in cardiomyocytes under normoxic conditions (Figure 7(a), before hypoxia).

However, both hypoxia and electrostimulation lead to increased ROS in control cells. Reoxygenation initially increases the levels of ROS in cardiomyocytes whereas about 1 min after the start of reoxygenation further increase in ROS is blunted regardless of cardiomyocyte electrostimulation. Perhaps, this reflects the recovery of the normal redox state in cardiomyocytes following oxygen availability. Overall increase in ROS achieved in control cardiomyocytes during hypoxia-reoxygenation in our experiments was about 3.5-fold.

In the presence of 30 nM Oxacom cardiomyocytes demonstrated no significant increase of ROS compared to control cells when subjected to hypoxia/electrostimulation. Specifically, Oxacom attenuated the accumulation of ROS during the period of hypoxia 6-fold. Similarly, during the first minute of reoxygenation Oxacom limited ROS accumulation; however, noticeable increase in ROS was observed at a later time with no evidence for saturation. Overall increase in ROS was about 2-fold in cardiomyocytes treated with Oxacom, which is 75% less than in control cardiomyocytes. Thus, in the presence of Oxacom the accumulation of ROS in cardiomyocytes during hypoxia was markedly attenuated and the latter effect could be related to the better performance of Ca-ATPases and, perhaps, NCX/Na/K-ATPase in these cells.

It is possible that Oxacom acts to maintain the thiol groups of SERCA2 and other Ca^2+^-transporters in reduced state and/or protects them through a reversible S-nitrosylation. It is known that cysteine residues of proteins located on the cytosolic side of sarcoplasmic reticulum membrane [20] may undergo redox modifications such as S-S bond formation, S-glutathionylation, and S-nitrosylation (SNO). Some of the cysteine residues are modified nonspecifically whereas S-nitrosylation or S-glutathionylation of other cysteines may change the functional activity of affected proteins including SERCA2, RyR2 Ca^2+^-channels, and L-Type Ca^2+^-channels [21, 22]. Because superoxide and nitric oxide are constantly formed in cardiomyocytes these substances seem to act as endogenous regulators of Ca^2+^ transporting proteins [21]. In the case of RyR2, the direct relationship between the number of S-nitrosylated thiol groups and Ca^2+^-channel activity was demonstrated [8]. Modification of 2 cysteines activates the channel; modification of 4 cysteines is accompanied by a moderate channel activation. S-nitrosylation of 11 cysteine residues (about 3 residues in each subunit) causes a significant activation of RyR2 whereas the modification of twice as many cysteines leads to irreversible channel activation.

Based on the current estimations of in vivo nitric oxide levels ranging from 100 pM or less up to 5 nM [23] one can assume that 30 nM Oxacom could release amounts of NO comparable to those produced by endogenous NO synthases and sufficient for protein S-nitrosylation. An alternative/additional pathway for Oxacom to influence SERCA2 activity in cardiomyocytes would be through the activation of cyclic GMP-dependent protein kinase (PKG) and phosphorylation of SERCA2 principal inhibitor phospholamban and, perhaps, other SERCA2 regulators, in particular, Hsp20 [24]. Our findings that 0.3 nM Oxacom was equally effective as 30 nM Oxacom in prevention of Ca^2+^ accumulation in hypoxic cardiomyocytes favor NO signaling pathway rather than the direct nitrosylation of the set of cellular proteins. Finally, the attenuation by Oxacom of ROS generation during the period of hypoxia suggests possible stimulatory effects of this compound on antioxidant defense systems of cardiomyocytes different from glutathione system. One possible scenario is that by direct scavenging of the hydroxyl and superoxide radicals NO could protect cardiac Na/K-ATPase from inactivation [25] and consequently prevent Ca^2+^ overload through NCX working in reverse mode. Outlined multiple possibilities warrant further studies to elucidate in detail the molecular basis of Oxacom action on Ca^2+^ transporters and ROS production; such experiments have already been initiated.

## 4. Conclusions

Obtained results demonstrate that Oxacom is an effective pharmacologic compound capable of reducing hypoxic contractures and promoting increase in the speed and extent of LV developed pressure recovery during reoxygenation. Its action to attenuate cardiac arrhythmias and to preserve the ability of the heart to reproduce high rate of contractions suggests that Oxacom can improve the function of calcium transport proteins in cardiomyocytes. This assumption was supported by direct experiments in isolated cardiomyocytes. Oxacom eliminated calcium transport alterations that took place in control experiments in the form of the slow Ca^2+^ removal from the sarcoplasm and delayed cardiomyocyte relaxation.

This corresponds well to the view that Ca^2+^ removal from the sarcoplasm by Ca^2+^-ATPases of the sarcolemma and sarcoplasmic reticulum (PMCA and SERCA2) as well as by NCX/Na/K-ATPase tandem is energy-dependent process that slows down due to the depletion of high-energy phosphates during the shortage of oxygen. However, contractile process requires much more energy than the work of the membrane ion pumps. In this regard, the prevention by Oxacom of hypoxic contracture and more rapid recovery of the contractile function during reoxygenation in the presence of this compound suggest protective effects on mitochondria. Observed action of Oxacom to limit ROS generation in hypoxic cardiomyocytes additionally supports the mitochondrial vector for the future mechanistic studies of this novel cardiovascular drug.

## Supplementary Material

Experimental data on Ca^2+^ transients in rat cardiomyocytes subjected to hypoxia-reoxygenation in the presence of 0.3 nM Oxacom.

## Figures and Tables

**Table 1:** Functional parameters of the isolated heart in the absence and in the presence of Oxacom in hypoxic perfusate.

	Before hypoxia		After hypoxia (30 min) and reoxygenation (30 min)	
	Control	Oxacom	Control	Oxacom
	(*n* = 15)	(*n* = 9)	(*n* = 11)	(*n* = 9)
Heart rate, beats/min	267 ± 9	250 ± 10	267 ± 11	253 ± 9
LV developed pressure, mm Hg	183 ± 6	195 ± 6	61 ± 8	79 ± 6
+*dP*/*dt*max, mm Hg/s	4490 ± 200	4830 ± 160	1520 ± 167	2020 ± 169
–*dP*/*dt*max, mm Hg/s	2630 ± 96	2670 ± 116	1240 ± 196	1760 ± 93^*∗*^
LV diastolic pressure, mm Hg	10 ± 1	9 ± 1	59 ± 5	45 ± 4
Coronary flow rate, mL/min	18.0 ± 1.7	18.1 ± 1.1	16.8 ± 4.3	13.3 ± 1.2

^*∗*^ *p* < 0.05 versus control after hypoxia-reoxygenation.
