# Supplementary material for: Protective Effects of Dinitrosyl Iron Complexes under Oxidative Stress in the Heart
Source: Oxid Med Cell Longev. 2017 Mar 21;2017:9456163. doi: 10.1155/2017/9456163 (PMC5379096; doi:10.1155/2017/9456163)
Supplement: Supplementary file 1 — Experimental data on Ca2+ transients in rat cardiomyocytes subjected to hypoxia-reoxygenation in the presence of 0.3 nM Oxacom. [file 9456163.f1.pdf]

Supplemental figures

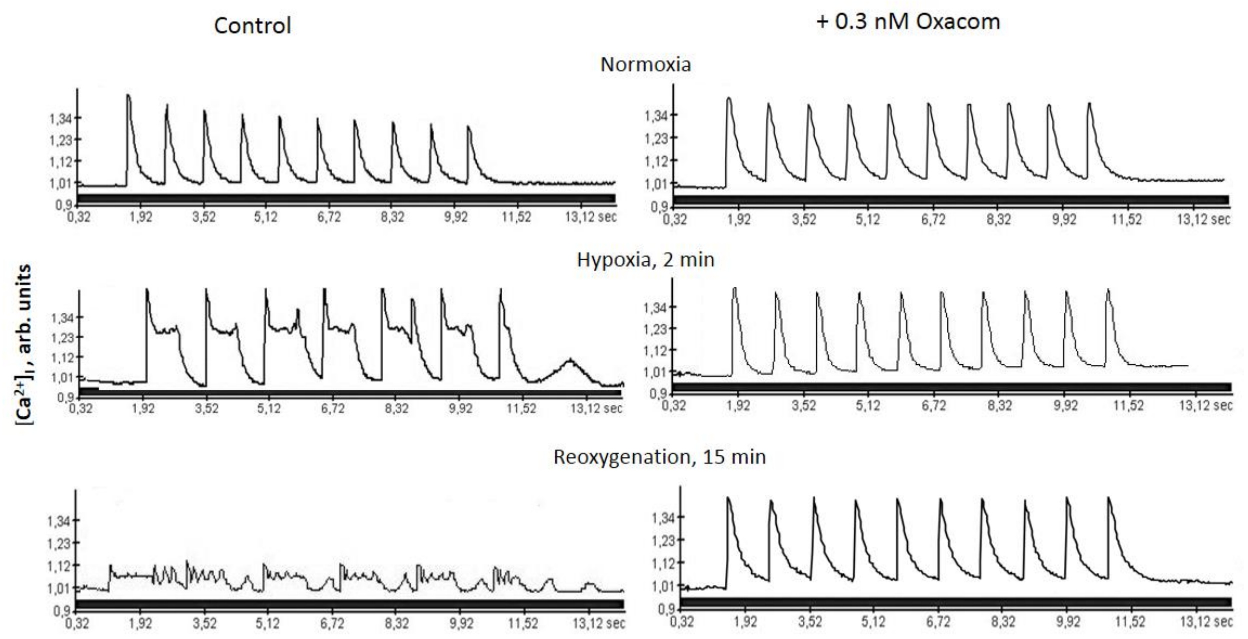

Figure S1. Effects of Oxacom on Ca^{2+} transients in isolated adult rat cardiomyocytes subjected to hypoxia-reoxygenation.

Cardiomyocytes loaded with fluo-4 were preincubated with 0.3 nM Oxacom for 30 min and electrostimulated by ten rectangular pulses at 1 Hz under conditions of normoxia, after 2 min of hypoxia produced by exchanging regular perfusion buffer saturated with carbogen (95% O_2, 5% CO_2) for hypoxic buffer saturated with 95% N_2, 5% CO_2; and then after 15 min of reoxygenation. Typical recordings are shown. The legend for ordinate on the left applies to all graphs.

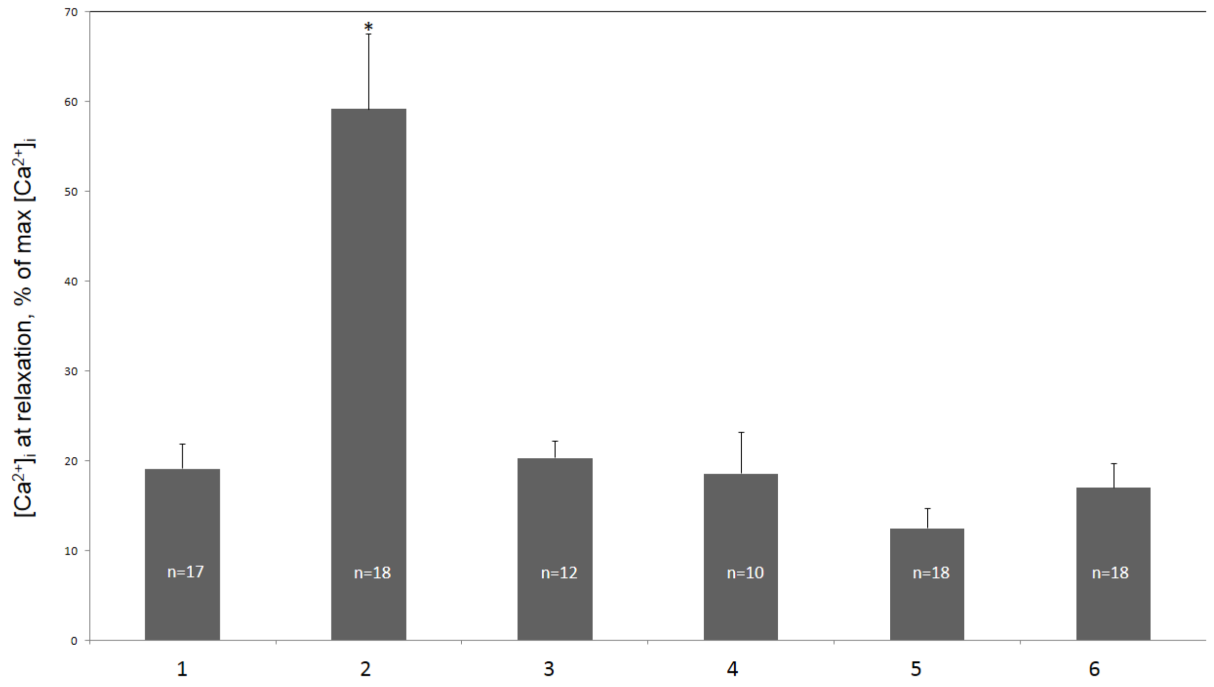

Figure S2. Quantitation of Oxacom effects on Ca^{2+} levels at relaxation in isolated adult rat cardiomyocytes subjected to hypoxia-reoxygenation.

1, control (normoxia); 2, hypoxia, 2 min; 3, hypoxia + 30 nM Oxacom, 2 min; 4, reoxygenation + 30 nM Oxacom, 15 min; 5, hypoxia + 0.3 nM Oxacom, 2 min; 6, reoxygenation + 0.3 nM Oxacom, 15 min. n, number of cells for each condition. Mean+s.d.

* $p<0.01$ 2 vs 1,3,4,5,6.
